# Myositis with prominent B-cell aggregates causing shrinking lung syndrome in systemic lupus erythematosus: a case report

**DOI:** 10.1186/s41927-021-00240-0

**Published:** 2022-02-16

**Authors:** Flavie Roy, Pat Korathanakhun, Jason Karamchandani, Bruno-Pierre Dubé, Océane Landon-Cardinal, Nathalie Routhier, Caroline Peyronnard, Rami Massie, Valérie Leclair, Alain Meyer, Josiane Bourré-Tessier, Minoru Satoh, Marvin J. Fritzler, Jean-Luc Senécal, Marie Hudson, Erin K. O’Ferrall, Yves Troyanov, Benjamin Ellezam, Jean-Paul Makhzoum

**Affiliations:** 1 Department of Medicine, Université de Montréal, Montreal, QC Canada; 2 Department of Pathology, Montreal Neurological Hospital and Institute, McGill University, Montreal, QC Canada; 3 Division of Pulmonary Medicine, Department of Medicine, Centre Hospitalier de l’Université de Montréal, Université de Montréal, Montreal, QC Canada; 4 Division of Rheumatology, Department of Medicine, Centre Hospitalier de l’Université de Montréal, Université de Montréal, Montreal, QC Canada; 5 Department of Medicine, CHUM Research Center, Université de Montréal, Montreal, QC Canada; 6 Division of Internal Medicine, Department of Medicine, Hôpital du Sacré-Coeur de Montréal, Université de Montréal, 5400 Gouin O Blvd, Montreal, QC H4J 1C5 Canada; 7 Division of Neurology, Department of Medicine, Hôpital du Sacré-Coeur de Montréal, Université de Montréal, Montreal, QC Canada; 8 Department of Neurology and Neurosurgery, Montreal Neurological Hospital and Institute, McGill University, Montreal, QC Canada; 9 Division of Rheumatology, Department of Medicine, Jewish General Hospital, McGill University, Montreal, QC Canada; 10 Faculté de médecine, Université de Strasbourg, Service de physiologie, explorations fonctionnelles musculaire, Service de rhumatologie et Centre de références des maladies autoimmunes rares, EA 3072, Hôpitaux universitaires de Strasbourg, Université de Strasbourg, Strasbourg, France; 11 Department of Clinical Nursing, School of Health Sciences, University of Occupational and Environmental Health, Kitakyushu, Japan; 12 Cumming School of Medicine, University of Calgary, Calgary, AB Canada; 13 Division of Rheumatology, Department of Medicine, Hôpital du Sacré-Coeur de Montréal, Université de Montréal, Montreal, QC Canada; 14 Department of Pathology, Centre Hospitalier Universitaire Sainte-Justine, Université de Montréal, Montreal, QC Canada

**Keywords:** Systemic lupus erythematosus, Dyspnea, Respiratory diaphragm, CD20 antigen, B cell, Ultrasound, Case report

## Abstract

**Background:**

Shrinking lung syndrome (SLS) is a rare manifestation of systemic lupus erythematosus (SLE) characterized by decreased lung volumes and diaphragmatic weakness in a dyspneic patient. Chest wall dysfunction secondary to pleuritis is the most commonly proposed cause. In this case report, we highlight a new potential mechanism of SLS in SLE, namely diaphragmatic weakness associated with myositis with CD20 positive B-cell aggregates.

**Case presentation:**

A 51-year-old Caucasian woman was diagnosed with SLE and secondary Sjögren’s syndrome based on a history of pleuritis, constrictive pericarditis, polyarthritis, photosensitivity, alopecia, oral ulcers, xerophthalmia and xerostomia. Serologies were significant for positive antinuclear antibodies, anti-SSA, lupus anticoagulant and anti-cardiolopin. Blood work revealed a low C3 and C4, lymphopenia and thrombocytopenia. She was treated with with low-dose prednisone and remained in remission with oral hydroxychloroquine. Seven years later, she developed mild proximal muscle weakness and exertional dyspnea. Pulmonary function testing revealed a restrictive pattern with small lung volumes. Pulmonary imaging showed elevation of the right hemidiaphragm without evidence of interstitial lung disease. Diaphragmatic ultrasound was suggestive of profound diaphragmatic weakness and dysfunction. Based on these findings, a diagnosis of SLS was made. Her proximal muscle weakness was investigated, and creatine kinase (CK) levels were normal. Electromyography revealed fibrillation potentials in the biceps, iliopsoas, cervical and thoracic paraspinal muscles, and complex repetitive discharges in cervical paraspinal muscles. Biceps muscle biopsy revealed dense endomysial lymphocytic aggregates rich in CD20 positive B cells, perimysial fragmentation with plasma cell-rich perivascular infiltrates, diffuse sarcolemmal upregulation of class I MHC, perifascicular upregulation of class II MHC, and focal sarcolemmal deposition of C5b-9. Treatment with prednisone 15 mg/day and oral mycophenolate mofetil 2 g/day was initiated. Shortness of breath and proximal muscle weakness improved significantly.

**Conclusion:**

Diaphragmatic weakness was the inaugural manifestation of myositis in this patient with SLE. The spectrum of myologic manifestations of myositis with prominent CD20 positive B-cell aggregates in SLE now includes normal CK levels and diaphragmatic involvement, in association with SLS.

## Background

Shrinking lung syndrome (SLS) is a rare respiratory manifestation of systemic lupus erythematosus (SLE), with an estimated prevalence of 1% [1, 2]. SLS is characterized by dyspnea, restrictive ventilatory defects on pulmonary function testing, small lung volumes on imaging and bilateral diaphragmatic weakness [2, 3]. Ultrasonography has been shown to be useful in assessing diaphragmatic weakness [4–7]. Current opinions on the pathophysiology of SLS in SLE center primarily around chest wall dysfunction secondary to pleuritis [8–10]. Other proposed mechanisms include bilateral phrenic neuropathy [11] and, although only a single muscle biopsy has been reported [12], inflammation of the diaphragm muscle [13]. We report a case of SLS in a patient with SLE and myositis; a novel finding was prominent CD20+ B-cell aggregates on muscle biopsy.

## Case presentation

In 2009, a 51-year-old Caucasian woman was diagnosed with SLE, secondary Sjögren’s syndrome and hypothyroidism. In the preceding months, she had pleuritis, polyarthritis, photosensitivity, alopecia, oral ulcers, xerophthalmia and xerostomia. Laboratory findings on admission demonstrated the presence of antinuclear antibodies (HEp-2 ANA, in a titer of 1/320 with speckled and nucleolar patterns), lupus anticoagulant, anti-cardiolipin, and anti-SSA autoantibodies. Hypocomplementemia (low C3 and C4), lymphopenia, thrombocytopenia and hypothyroidism (TSH > 100 mU/L) were also documented. She was admitted to the hospital for progressive dyspnea and anasarca. Cardiac ultrasound showed a normal left ventricular ejection fraction, no pericardial effusion but elevated systolic pulmonary artery pressure (49 mmHg). Pericardial calcifications and thickening were detected by computed tomography. On cardiac catheterization, mean pulmonary artery pressure was 28 mmHg and wedge was 13 mmHg. Diuretic therapy led to a weight loss of 12 kg and normalisation of the pulmonary arterial hypertension on repeat cardiac catheterization. A diagnosis of constrictive pericarditis was made. Thyroid hormone supplementation was initiated. SLE manifestations responded to low-dose corticosteroids and hydroxychloroquine 200 mg twice daily.

**Fig. 1:**
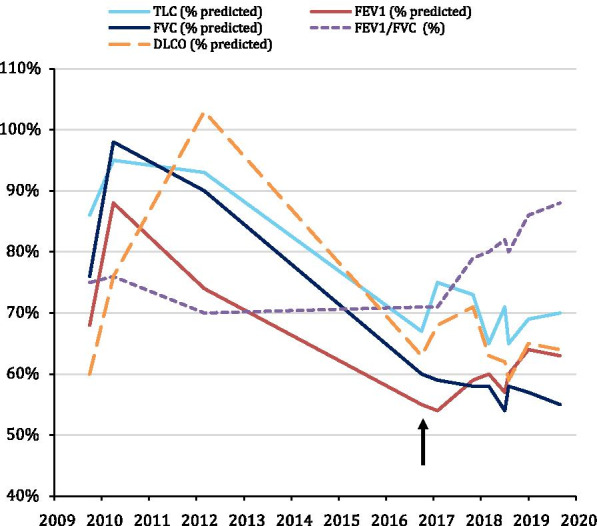
Course of pulmonary function tests over time. Course of pulmonary function tests over time, showing improvement with onset of prednisone and mycophenolate mofetil (arrow). *TLC* total lung capacity, *FVC* forced vital capacity, *DLCO* Diffusing capacity of the lung for carbon monoxide, *FEV1* forced expiratory volume in 1 second

In 2016, she experienced new-onset Raynaud’s phenomenon, mild proximal muscle weakness, and progressive exertional dyspnea. The patient’s exam demonstrated moderate symmetric muscle weakness in axial, hip and shoulder girdle muscles. Weakness was graded at 4− to 4+ out of 5 on the MRC scale for neck extensors, shoulder abductors, internal and external rotation of the shoulders, flexion and extension of the elbows and movements of the hip (flexion, abduction, adduction, extension). There was also some mild weakness (4+ out of 5) for finger extension and finger abduction. Serial pulmonary function testing revealed a progressive restrictive ventilatory defect (Fig. 1). On March 2018, while on oral hydroxychloroquine alone, pulmonary function testing showed a marked restrictive pattern: total lung capacity was 2.90 L (65% of the predicted normal value), forced expiratory volume in 1 second (FEV_1_) was 1.36 L (60%), and forced vital capacity (FVC) was 1.70 L (58%). Diffusing capacity of the lung for carbon monoxide (D_L_CO) was 63% of the predicted value. In the supine position, FEV_1_ and FVC both declined by 10%. Maximal inspiratory pressure was 109% of predicted. Chest radiograms revealed chronic elevation of the right hemidiaphragm. Chest computed tomography showed no evidence of interstitial lung disease, and cardiac ultrasound and ventilation-perfusion scans were normal.

**Fig. 2:**
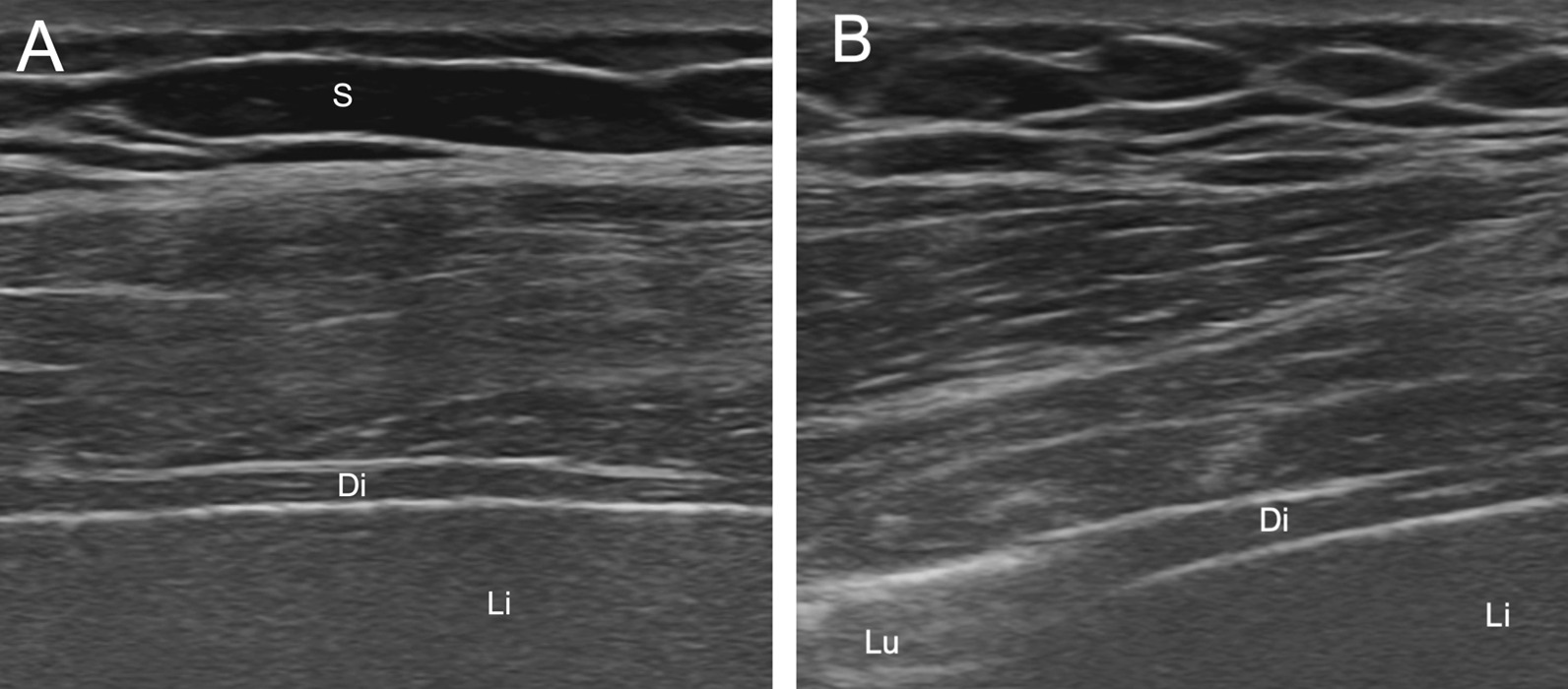
B-mode ultrasound of the right hemidiaphragm. **A** ultrasound performed during end-expiration. **B** Ultrasound performed during maximal inspiration. The diaphragm is visible between two-hyperechoic lines representing the pleural and peritoneal membranes, respectively. At the end of maximal inspiration, lung tissue is visible in **B**, end-expiration diaphragm thickness was 1.90 mm. *Di* diaphragm, *S* subcutaneous tissue, *Li* liver, *Lu* lung

Diaphragmatic ultrasound was performed in July 2018 to assess diaphragmatic contractility. Right and left diaphragm thickening fractions (TFdi) were respectively of only 38% and 45% (mean normal TFdi values in the healthy population are approximately 80%, with TFdi values < 20% being indicative of significant diaphragmatic weakness) [4] (Fig. 2). In the combined presence of unexplained dyspnea, an extrapulmonary restrictive lung disease, a positional decrease in FVC and TFdi values that were judged to be on the lower end of the normality spectrum (although no previous examination was available for comparison), a diagnosis of SLS was made [14].

**Fig. 3:**
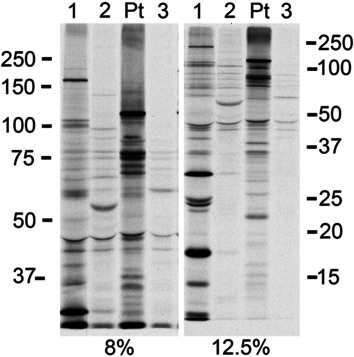
Immunoprecipitation using radiolabeled K562 cell extracts. ^35^S-methionine labeled human K562 cell lysates were immunoprecipitated by patients’ IgG and resolved by electrophoresis on 8% and 12.5% gels as previously described [25]. The 12.5% gels are used to provide better resolution of lower molecular mass proteins. The case report patient (Pt) is shown with comparator human sera bearing autoantibodies to U1RNP and survival of motor neuron (SMN) complex (lane 1), or anti-Ro60 (lane 2), and a negative human serum that did not immunoprecipitate any protein (lane 3)

Since the patient had a two-year history of Raynaud’s phenomenon with proximal and neck flexor muscle weakness on physical examination, additional investigations were undertaken. Serological markers of lupus showed normal dsDNA, but low complement levels. Nailfold capillaroscopy as well as serum creatine kinase (CK) and aldolase levels were normal. Serology demonstrated the presence of anti-Ro-52/TRIM21 (high positive), but no myositis-specific, SSc-specific or SSc-overlap autoantibodies were found on line immunoassays (Euroimmun AG, Luebeck, Germany) or on Protein A-assisted immunoprecipitation using radiolabeled K562 cell extracts (Fig. 3). A muscle MRI of the lower limbs was unremarkable.

**Fig. 4:**
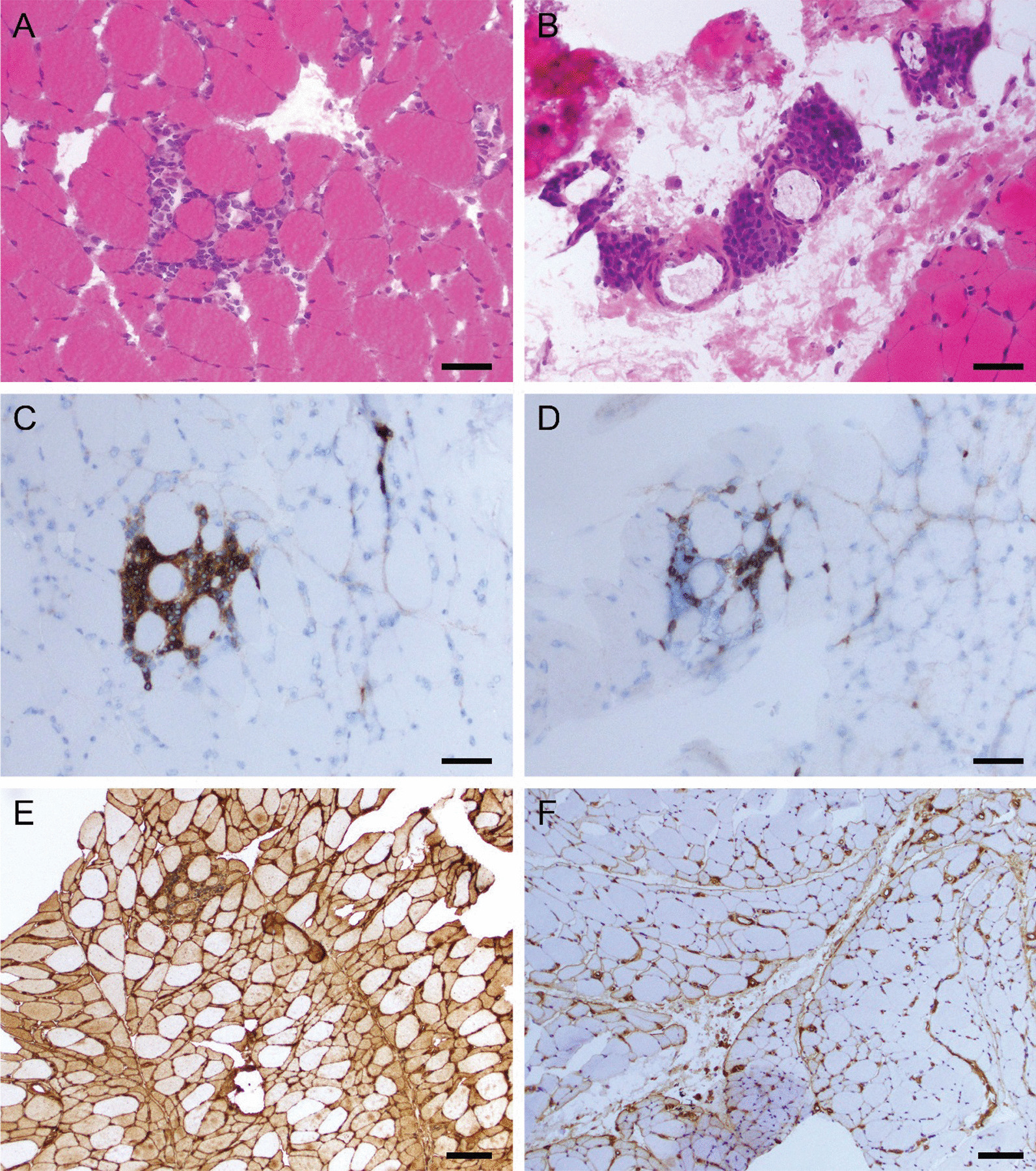
Muscle biopsy findings. **A**, **B** Hematoxylin & eosin staining showing dense endomysial lymphocytic aggregates (**A**), and perimysial fragmentation with plasma cell-rich perivascular infiltrates (**B**). **C**–**F** Immunohistochemical stains showing endomysial aggregates composed of predominant CD20 + B cells (**C**) and accompanying CD3 + T cells (**D**); diffuse upregulation of MHC class I (**E**); and perifascicular upregulation of MHC class II (**F**). *Bar*, **A**–**D** 50 μm (× 20 objective); **E**–**F** 100 μm (× 10 objective). Method for picture acquisition: Olympus BX46 microscope, with UPlanFL objectives with CellSens software (Olympus). The six images were placed in photoshop, in a 600dpi canvas, without further processing, to organize them and include lettering and bars. The final panel was exported as a jpg with 80% quality setting

Electromyography revealed fibrillation potentials in the biceps, iliopsoas, cervical and thoracic paraspinal muscles, and complex repetitive discharges in cervical paraspinal muscles. In addition, myopathic units (small amplitude, polyphasic motor unit potentials) in triceps, first dorsal interosseous and vastus lateralis were observed. Biceps muscle biopsy revealed dense endomysial lymphocytic aggregates rich in CD20+ B cells, perimysial fragmentation with plasma cell-rich perivascular infiltrates, diffuse sarcolemmal upregulation of class I MHC, perifascicular upregulation of class II MHC (Fig. 4), and focal sarcolemmal deposition of C5b-9 (not shown), meeting the diagnostic criteria of polymyositis by the 119th ENMC classification criteria [15].

Treatment with prednisone 15 mg/day and mycophenolate mofetil 2 g/day was initiated, and shortness of breath and proximal muscle weakness improved. In March 2019, diaphragmatic ultrasonography revealed marked improvement in diaphragmatic function: right TFdi was 51% and left TFdi 128%. Pulmonary function testing also showed improvement of the restrictive ventilatory defect: total lung capacity was 3.13 L (70% predicted), FEV1 was 1.41 L (63% predicted), FVC was 1.61 L (60% predicted) and D_L_CO was stable.

## Discussion and conclusions

This is the first report of myositis with diaphragmatic weakness, normal CK levels and prominent B-cell aggregates on muscle biopsy in a patient with SLE and SLS. The clinical presentation and the improvement of muscle strength and pulmonary function tests after immunosuppressive therapy suggests diaphragmatic myositis with B cell infiltrates as a cause of SLS in SLE.

In 1984, polymyositis presenting with respiratory failure secondary to diaphragmatic weakness was reported by Blumbergs et al. [16]. The case was noteworthy for dyspnea as the initial symptom with brachial as well as neck flexor and extensor weakness on examination. A quadriceps muscle biopsy showed endomysial lymphocytic inflammation, and autopsy of the diaphragm and the intercostal muscles demonstrated lymphocytic inflammation, with associated plasma cells and macrophages. Unfortunately, CD20 staining was not reported. Since then, larger series have reported diaphragmatic involvement in myositis [17], including diaphragmatic involvement in immune checkpoint inhibitor-related myositis [18]. In fact, autopsy findings of a patient presenting with normal CK levels and isolated diaphragmatic weakness was noteworthy for CD4 positive and CD8 positive infiltrates on examination of the diaphragm and intercostal muscles [19].

SLS has been associated with SLE, but was also reported in Sjögren's syndrome [20], rheumatoid arthritis [21] and systemic sclerosis (SSc) [22]. A recent case series on SLS in SLE excluded patients with overt myositis [2]. The first reported autopsy study of the diaphragm in a patient with SLS and SLE demonstrated diffuse fibrosis and diaphragmatic muscle atrophy [13]. Subsequently, muscle biopsy of a patient with myositis, SLE and diaphragmatic weakness showed CD4-predominant endomysial lymphocytic infiltrates in the deltoid [12], but CD20 staining was not reported.

We report for the first time a patient with SLE and secondary Sjögren's syndrome who presented with SLS as the inaugural manifestation of a myositis with normal CK levels. Hypothesis for normal CK include muscle fiber inflammation in the absence of muscle fiber necrosis, predominant involvement of intramuscular capillaries, or when atrophy occurs in the setting of a chronic myositis. Muscle biopsy of the biceps showed myositis with prominent endomysial CD20+ B-cell aggregates, and perimysial inflammation with fragmentation. These muscle pathology findings are similar to a description of 10 patients with brachio-cervical inflammatory myopathies, three of whom had respiratory weakness [23]. Interestingly, rituximab, a monoclonal antibody against the CD20 protein, has emerged as an efficacious therapy for shrinking lung syndrome in SLE [3, 24–26], suggesting that some patients with SLS may have undiagnosed diaphragmatic myositis with prominent CD20+ B cell infiltrates.

In conclusion, diaphragmatic weakness was the inaugural manifestation of myositis in this patient with SLE. The spectrum of myologic manifestations of myositis with prominent B-cell aggregates in SLE now includes normal CK levels and diaphragmatic involvement, in association with SLS.

## Data Availability

The datasets used and/or analysed during the current study are available from the corresponding author on reasonable request.

## Abbreviations

ANA: Antinuclear antibody
CK: Creatinine kinase
DLCO: Diffusion capacity of the lungs for carbon monoxide
FEV1: Forced expiratory volume in 1 second
FVC: Forced vital capacity
HEp-2: Human epithelial type 2 cell
IgG: Immunoglobulin G
MHC: Major histocompatibility complex
SLS: Shrinking lung syndrome
SLE: Systemic lupus erythematosus
SMN: Survival motor neuron
Anti-SSA: Anti-Sjögren’s-syndrome related antigen A autoantibodies
SSc: Systemic sclerosis
TLC: Total lung capacity
TFdi: Diaphragm thickening fraction
TRIM21: Tripartite motif-containing 21
TSH: Thyroid stimulating hormone
